# Adapting an evidence-based HIV behavioral intervention for South African couples

**DOI:** 10.1186/s13011-015-0005-6

**Published:** 2015-02-24

**Authors:** Wendee M Wechsberg, Nabila El-Bassel, Tara Carney, Felicia A Browne, Bronwyn Myers, William A Zule

**Affiliations:** Substance Abuse Treatment Evaluations and Interventions Research Program, RTI International, Research Triangle Park, NC USA; Gillings School of Global Public Health, University of North Carolina, Chapel Hill, NC USA; Psychology in the Public Interest, North Carolina State University, Raleigh, NC USA; Psychiatry and Behavioral Sciences, School of Medicine, Duke University, Durham, NC USA; Columbia University School of Social Work, New York, NY USA; Alcohol, Tobacco and Other Drug Research Unit, South African Medical Research Council, Parow, Cape Town, South Africa; Harvard School of Public Health, Boston, MA USA; Department of Psychiatry and Mental Health, University of Cape Town, Cape Town, South Africa

**Keywords:** HIV, Heterosexual couples, Intervention adaptation, Substance abuse, Gender-based violence, Sex risk behaviors, South Africa

## Abstract

**Background:**

In South Africa, heterosexual couples are at risk for HIV infection and transmission through substance use, gender-based violence and traditional gender roles, and sex risk behaviors such as having multiple partners and unsafe sex.

**Methods:**

To address these interconnected HIV risks among heterosexual couples, we used the ADAPT framework to modify an existing, efficacious women’s HIV prevention intervention (the Western Cape Women’s Health CoOp) to include components of an evidence-based couple’s intervention from the United States (Project Connect) and components from the Men as Partners program that has been used successfully in South Africa. We conducted focus groups with men, women and couples, and obtained feedback from a long-standing Community Collaborative Board (CCB) to guide the synthesis of elements of these three interventions into a new intervention. We then piloted the adapted intervention for feasibility and acceptability.

**Results:**

The new intervention is called the Couples’ Health CoOp. This intervention targets men who use alcohol and other drugs and engage in unprotected sex, and their main female sex partners. The intervention addresses substance use, sex risk, HIV and other sexually transmitted infections, gender roles, gender-based violence, communication skills, and goal-setting activities to increase sexy (eroticize) safe-sex behaviors. The Couples’ Health CoOp also includes “voices” from the focus group members to ground the intervention in the experiences of these at-risk couples. In addition, it utilizes a participant handbook that reiterates workshop content and includes homework assignments for couples to complete together to increase problem-solving skills within their relationship, and to improve their sexual relationship and help sustain HIV risk-reduction strategies. All of these adaptations were based on participants’ suggestions made during formative work and pilot testing.

**Conclusions:**

The Couples’ Health CoOp is a couple-based HIV prevention intervention that targets alcohol and other drug use to reduce sexual risk, reduce gender-based violence and offer alternatives for conflict resolution, promote healthy relationships, and modify traditional gender roles in South Africa.

**Trial registration number:**

NCT01121692.

## Background

HIV continues to be a major public health problem in South Africa, especially among women [1,2]. While HIV prevalence among South Africans is estimated to be 12.2%, the estimated prevalence among women of reproductive age is 17.3%, with HIV incidence being significantly higher among women than men [3]. In poor communities in South Africa, women who are in relationships are more likely than their male partners to be infected with HIV [4]. Even in areas where condoms are highly accessible, condom use is low among heterosexual couples, with only about 12% of South African couples reporting consistent condom use [5]. The high rates of HIV indicate a need for innovative HIV prevention interventions that address risk behaviors that occur within the context of heterosexual couples and increase the risk for HIV acquisition and transmission.

While men are generally seen as the source of HIV transmission in heterosexual relationships, especially among sero-discordant couples, evidence suggests that both partners engage in risky behaviors [6]. Recognition is increasing that HIV positive women in discordant couples may have acquired HIV infection, from a previous partner or from a current partner outside the couple, and in these cases women may transmit HIV to their primary male partner. Prior research has identified several factors that put women at risk for HIV within the context of their sexual relationships with men. First, substance use is prevalent in Western Cape communities and it has been linked to HIV transmission through its association with unprotected sex and other risk behaviors [7]. Specific substances such as methamphetamine [8,9] and alcohol [9] have especially been linked to risky sex behaviors. Both the male and female partners in heterosexual relationships can also be vulnerable to HIV transmission through multilevel risk environments, as substance use appears to negatively impact their relationships. For example, substance use is associated with concurrent sex partners among men [10], survival sex among women when their partners spend money on substances rather than providing for their household, and increased risk of victimization [10]. The significant role of substance use in social and sexual interactions in some South African townships [8] and its association with impaired judgment and risky decision-making [11-13] creates even greater obstacles for women to negotiate the conditions of sex and to reduce their risk for HIV [14,15]. These conditions speak to the urgency of addressing HIV prevention needs within heterosexual couples in South Africa [16-19].

Second, micro-level factors also influence risk for HIV. Intimate partner violence (IPV) is common in heterosexual relationships in South Africa [20,21]. IPV constrains women’s ability to negotiate condom use (for fear of violence) and often takes the form of forced sex [21,22], which further fuels women’s risk for HIV within the context of relationships [20,23]. Relationship conflict that leads to IPV [9,10,24,25] is often underpinned by jealousy and substance use. While women-focused interventions can help women reduce their substance use risks for HIV [26], in order to produce effective and sustainable changes in the risk environment for vulnerable women, it is critical that HIV risk-reduction interventions also engage male partners [10,27] and address risk that occurs within the couples’ context.

Third, on the macro level, the communities in which couples live may increase their HIV risk. In many communities, traditional sex roles, cultural acceptance of multiple sex partners for men [24], gender inequality, and limited interpersonal communication are widespread and increase the probability of high-risk sex activities within relationships. Previous qualitative research in Cape Town has shown that it is acceptable for men who are in a relationship to have multiple sex partners, but it is not acceptable for their female partners [10,27]. In poor South African townships, many couples socialize in drinking venues known as “shebeens.” Patronage of these venues by one or both members of the couple increases HIV risk, as these drinking venues are associated with alcohol and other drug use, high-risk sexual behavior, and violence [28].

In addition, couple-based approaches to HIV prevention acknowledge women’s unique social, cultural, and biological vulnerabilities and susceptibility to HIV; that men and women can infect each other; and that couples can reduce their HIV risks by working together [19]. Empowering women, working with men to shift gender imbalances, and strengthening couples’ communication and conflict resolution skills have all been identified as key components for reducing HIV risk within couples [29-32], but these essential components are rare in most HIV prevention interventions [33,34].

Despite evidence that interventions with couples are an important component of effective HIV prevention programs [35], very few HIV risk-reduction interventions target heterosexual couples from communities in South Africa [36] with high HIV prevalence and where one or both partners report substance use. This article describes the process of adapting an efficacious, evidence-based intervention (EBI), the Women’s Health CoOp (WHC), to address the HIV prevention needs of South African couples. This adaptation involved integrating components of Project Connect (a best-evidence HIV behavioral intervention for couples) and the Men as Partners (MAP) program [37] (an intervention designed to engage men in reducing gender-based violence) into the WHC to comprehensively address the interrelated issues of substance use, sex risk behaviors, gender role expectations, and gender-based violence within the context of couples’ primary relationships.

### The primary intervention of origin: WHC in South Africa

The original Women’s CoOp (WC), a women-focused intervention consisting of two sessions designed to decrease substance use and sex risk, was developed in the United States in North Carolina with African American women who use drugs [38]. It has been classified by the U.S. Centers for Disease Control and Prevention (CDC) as a “best-evidence” behavioral intervention for HIV prevention [39]. Its seven core elements, which are based in feminist and empowerment theories, include (1) educational cue cards that address risk-reduction information for substance use and HIV and other sexually transmitted infections (STIs), (2) peer interventionists who receive extensive training, (3) behavioral skills training for sexual protection, (4) role-plays for how to negotiate safer sex and how to communicate, (5) individualized action plans to set goals and strategies for behavior change, (6) HIV testing, and (7) referral to necessary agencies [40]. The core elements of the original intervention have been adapted for a variety of settings, including for college women in the United States [41], pregnant women in the United States [26] and South Africa [42], and substance-using sex workers and other vulnerable drug-using women in South Africa [43,44] and Russia [45].

In the South African adaptation, the WHC modules on victimization and violence prevention were developed, tested, and found to be efficacious [26]. These modules included content on dealing with violent men, risks associated with dry sex, rape issues, and violence prevention [15]. The WHC was also adapted for use with drug-using women in the Western Cape, and called the Western Cape Women’s Health CoOp (WCWHC). This intervention has proven to be efficacious in reducing HIV risk behavior in this population [44].

### Evidence-based elements from a couples-based study: Project Connect

The CDC has identified Project Connect as a “best evidence” HIV behavioral intervention for couples [39]. It was initially developed for heterosexual couples in the United States who are at risk for HIV and other STIs. The intervention is based on the AIDS-Risk Reduction Model and Ecological Perspective [35]. It consists of an orientation session and five relationship-based sessions with both the male and female partners. It emphasizes the importance of communication within relationships (i.e., the Speaker-Listener technique), negotiation and problem-solving skills, help-seeking and social-support skills, couples testing together for HIV and linkages to HIV and other services, gender roles and expectations around safer sex and partner abuse, how to communicate and share safer sex practices with other couples and their social network, and how to have safe and fun sex (i.e. eroticize safe sex) choosing from the Connect Sex Café [35]. Project Connect has been shown to be efficacious in reducing HIV risk behavior [35,46]. It has been adapted for methamphetamine-using men who have sex with men in the United States [47], as well as for people in the United States and internationally who inject drugs [48,49].

### Elements from the Men as Partners program

The original format of the Men as Partners (MAP) program was developed by EngenderHealth and the Planned Parenthood Association of South Africa. It was based on an ecological framework [37]. The curriculum, which lasts up to 5 days [50], is designed to engage men in reducing gender-based violence by challenging men’s attitudes, values and behaviors, and promoting positive sexual and reproductive health, including the prevention of HIV/AIDS. Although it is not an EBI, MAP has been used extensively in South Africa [37]. In its original format, however, it does not address substance use as a risk behavior for gender-based violence or HIV [50]. Several of the components around gender roles and activities were selected from the original program and adapted for the new intervention.

## Methods

The foundation of the new couples’ intervention is the WCWHC intervention, as it addresses the intersection of HIV, sex risk behaviors, alcohol and other drug use, and gender-based violence in the context of South Africa; has demonstrated efficacy in addressing these multiple risk behaviors among different populations of women in South Africa, and has cultural congruence for people from poor communities in the Cape Town area. The couples’ intervention also incorporates elements from the Project Connect intervention and the MAP program.

We applied the ADAPT framework [51] through a systematic and iterative process to identify gaps in the WCWHC intervention as well as the elements from the Project Connect intervention and the MAP program that could be used to fill these gaps. We then adapted the WCWHC intervention and elements from the other interventions. Finally, we synthesized all of the elements to create a new intervention for use with heterosexual couples. The primary objective of the adaptation process was to ensure that the new intervention addressed substance use, gender-based violence, and sex risk for HIV within the context of South African couples who frequent shebeens, in township communities, and also to strengthen couples’ problem-solving and communication skills as well as increase their desire for a monogamous relationship by promoting safe but exciting sex.

The ADAPT framework is widely used to adapt EBIs for new contexts while maintaining the core elements of the original intervention. It has been used extensively in the adaptation of HIV prevention programs [51]. The framework comprises the following steps: (1) Assessment; (2) Making decisions on adopting, adapting, or selecting another intervention; (3) Adaptation of the Intervention; (4) Production; and (5) Topical Experts. A description of the methods followed in each step of the adaptation is shown in Table 1.

**Table 1:** **Adaptation steps, methods, and intervention revisions**

**Step**	**Method**	**Version of interventions**
Assessment	Assess new target population: heterosexual couples by conducting 10 focus groups with female partners (n = 10), male partners (n = 21), and couples (n = 36)	Published findings^9^
Decision	Use assessment findings to inform decisions about how to adapt and synthesize WCWHC, MAP, and Project Connect for this population	WCWHC, MAP, and Project Connect
Administration/Pilot test	Administer the new CHC intervention in two focus group sessions	Revision 1
Production	Produce further revisions of the CHC intervention, incorporating pilot test feedback	Revision 2
Topical experts	Collect feedback from community collaborative board and developers of project connect and WCWHC and make further revisions to the CHC intervention	Revision 3

Note: *CHC* Couples’ health coop, *MAP* Men as partners program, *WCWHC* Western cape women’s health coop.

### Step 1: assessment

The assessment process involved assessing the need for the proposed intervention and determining which intervention components were relevant and acceptable to high-risk couples. To accomplish this, we had already conducted 10 focus groups—2 with women only (n = 10), 4 with men only (n = 21), and 4 with couples (n = 36)—to obtain feedback regarding intersecting risk behaviors and the intervention’s core elements and the feasibility of implementation of the Couples’ Health CoOp (CHC). The findings from this formative research have been published elsewhere [10].

The focus group facilitator presented the key components of WCWHC and selected elements from Project Connect and the MAP program, and asked for feedback on acceptability of and satisfaction with the content. Levels of knowledge regarding interrelated substance use and sexual and IPV risks for HIV were also explored. The focus groups were transcribed and coded to identify salient themes related to intervention needs.

Our long-standing Community Collaborative Board (CCB) actively participated in all stages of the adaptation process. Since 2005, this CCB has been a collaborative partner on several HIV prevention studies and has helped inform and shape how we adapt and implement interventions in this context. The CCB comprises representatives from non-governmental organizations, community-based organizations, government and research/academic institutions, and community members where the intervention will be tested once the adaptation process is completed. The CCB advised the researchers about issues in these communities and helped to build links to referral services for couples, such as social services. In addition, a separate expert panel was held with professionals with specific expertise in substance use, HIV, and interpersonal violence to obtain their input on what the intervention should address. This article focuses on ADAPT Steps 2 through 5.

### Step 2: decisions

We used the assessment findings to inform decisions about how to adapt and synthesize elements of each of the three interventions into the new CHC intervention. The Principal Investigator, South African Co-Investigator, Project Director, and Project Coordinator were all involved in the assessment step and are experienced in adapting EBIs for new contexts. Based on the findings, we made decisions about which of the main elements from the WCWHC would be retained as well as which components of Project Connect and the MAP program would be adapted to increase their relevance for heterosexual couples in the targeted neighborhoods [4]. We also decided which activities needed to be added to the CHC intervention to ensure that it was locally relevant for reducing HIV risk among couples.

**Figure 1:**
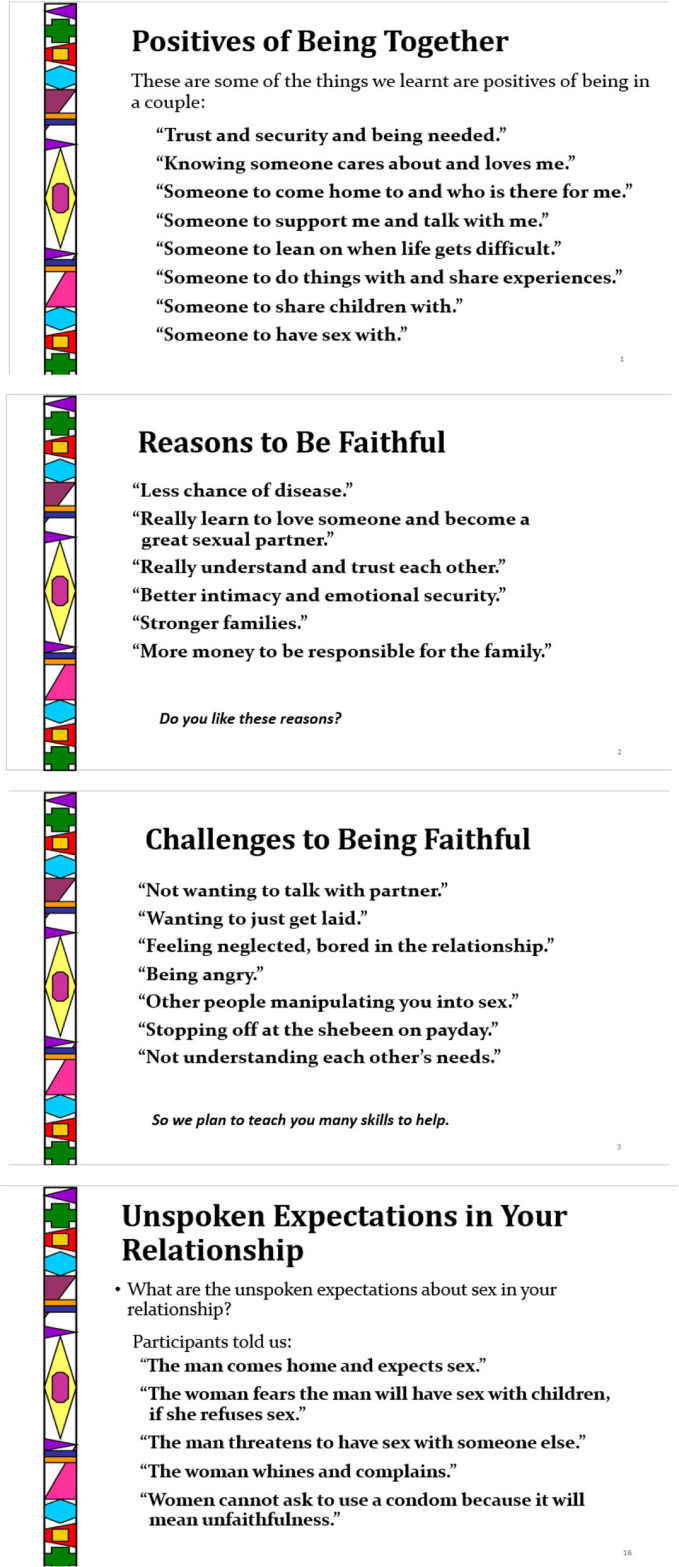
**Examples of the voices from focus group participants.**

These decisions included integrating the voices of male and female participants from the earlier focus groups into the intervention slides [10] to lend credibility and local relevance to the adapted CHC intervention (shown in Figure 1); incorporating more material on the interrelationships among substance use, risky sex and IPV and how these increase risk for HIV, and revising the terminology and visuals used in the intervention to make the intervention more culturally relevant to couples from impoverished Cape Town communities. In previous studies, participants reported that including the voices of participants from earlier formative stages helped newly engaged members feel that they could relate to the subject matter and begin a dialogue on these issues [26]. One of the most important aspects of Project Connect that was integrated into the new CHC intervention was communication skills activities, including the Speaker-Listener technique and problem-solving skills. This included two DVD clips of couples role-playing these skills. Very user-friendly, portable, battery-operated DVD players were used by facilitators to play these clips for workshop participants.

The new CHC intervention included a focus on communication and negotiation skills as well as the effect that traditional gender roles and expectations may have on keeping couples in conflict. It also incorporated how couples talked about these issues in their own voices from the earlier focus groups. As the intervention progresses, couples practice these exercises in dyads as well as the larger intervention group. Specifically, the intervention requires couples to participate in group exercises where they use a small portable white board to list reasons why couples stay together and what constitutes “good” or “bad” sex. Other activities from Project Connect require couples to talk privately with each other about why they value their relationship and what they value in their partner in order to practice their newly learned communication skills and be able to actualize a commitment pledge of fidelity. The final closing of the program offers information about community strengths and resources, and how couples who receive the intervention could act as role models for others. The intervention is designed to be delivered as two workshop sessions, with each workshop divided into two modules.

### Step 3: administration/pilot study

The pilot study phase included a final set of focus groups in which the CHC intervention was delivered to focus group participants. The target population was recruited through male partners who used alcohol and were in a primary sexual relationship for at least one year. Males and their female partners were screened for study eligibility separately but at the same time to avoid coercion. Upon completion, participants received a grocery voucher worth 50 ZAR (5 US dollars) as well as a health risk kit consisting of toiletries, condoms and lubricants. We conducted two focus groups with six couples (n = 12). The adapted CHC intervention was pilot tested over 2 days.

The purpose of this pilot test was to assess participants’ initial responses to the adapted and completed intervention, to obtain feedback on the intervention content, and to pilot the new activities and the skill-building and goal-setting components in order to determine how best to conduct the exercises within a group context. The pilot test also provided opportunities to observe the flow and monitor the timing of the different modules, and to assess the time required to deliver the entire intervention and instructions for activities so that any problems could be identified and rectified. As part of the evaluation of the pilot, participants were asked if they understood the content of the slides, if the language was appropriate, and if they learned anything new from the intervention. Participants were also asked to provide feedback on the visuals and DVD clips contained within the intervention, and on their experience of the activities. This pilot test of the intervention was delivered in local community centers.

### Step 4: production (results from the pilot test)

Following the pilot test, the project team reviewed the findings and subsequently made further adaptations to the intervention. First, based on participants’ suggestions, visuals were added to illustrate important concepts. For example, pictures of STIs were added. New slides were developed with pictures of shebeens and couples in South Africa. Second, the language used in the slides that originated from the WCWHC was modified to address both partners. As the duration of the couples’ intervention was much longer than expected, the project team incorporated participants’ suggestions about where changes could be made to reduce certain content, such as only showing two Project Connect video clips instead of three.

Following the pilot test and based on participants’ suggestions about the need for reference materials for the lessons learned, the project team developed a handbook for couples that reiterated the core elements of the CHC intervention and contained activities in which couples could practice together the skills taught during the intervention. The new intervention retained the Speaker-Listener technique and problem-solving components of Project Connect, in addition to a commitment pledge for monogamy. The intervention also ensured that important local traditions, such as the importance of family and children, were respected and promoted as strengths in these communities. All participants felt that the interventions would be accepted by other couples in their communities, although female participants reported that getting their male partners to attend the intervention may be “challenging”. Community centers in their neighborhoods were suggested as readily accessible places where it would be feasible to conduct the intervention. Participants were also in agreement that other couples would want to attend the couples’ intervention, to learn new skills that would benefit or “help them” in their relationship (such as communication and problem-solving skills) and then “pay it forward” (act as role models to other members of their community).

All of the intervention materials were presented in Microsoft PowerPoint and loaded onto a laptop computer for portability, and also printed out and placed into a flipchart booklet for delivery. Materials also included two video scenarios illustrating the Speaker-Listener technique from Project Connect, which participants reacted positively to. These videos can be loaded as video files on a computer, or they can be used to create a DVD that can be played on a portable DVD player or a computer with a built-in DVD player.

### Step 5: topical expert reviews and community collaborators

After adapting and producing the next version of the intervention and the accompanying materials, the next step involved program developers of Project Connect and the WCWHC reviewing the new CHC intervention and making further refinements. CCB members were also asked to comment on the handbook that was developed for the CHC intervention, as well as practical issues related to implementing the intervention. Further minor refinements were made based on the feedback of these topical experts. Table 2 presents an overview of the final adapted intervention. The table explicates which interventions influenced the adaptation and which components were entirely new based on the earlier focus groups including using their voices, pictures of local shebeens and other issues that became apparent in the process (e.g., building on strengths of family, development of the couples handbook).

**Table 2:** **Overview of intervention content for the couples health coop**

Workshop 1	•Welcome and introductions
Module 1	•Icebreaker-M
	•Reaching couples for HIV-W
	•Couple’s risk of HIV-W
	•Shebeens**
	•Interrelated risk for partners and HIV**
	•Linked behaviours-W
	•Circle of safety**
	•Activity: Positives of being a couple**
	•Reasons on being faithful**
	•A new way to communicate-C
	•Speaker-listener technique (and activity)-C
	•Substance use in South Africa –W 15 slides
	•Having a balanced life-W
	•Healthy social time**
	•Alcohol harm reduction-W
	•Benefits of substance use treatment-W
Workshop 1	•Interrelated risk for HIV-W
Module 2	•Couples and HIV-W
	•HIV facts-W
	•Window period-W
	•HIV infection and prevention-W
	•HIV testing-W
	•Other sexually transmitted injections (STIs)-W with 9 pictures**
	•Circumcision and decreasing HIV risk-*
	•Keeping private parts healthy-W
	•Reducing sex risks and condom use-W
	•Activity: condom steps-W
	•Male and female condoms-W-16 slides
	•Oral sex and protection-W-2 slides
	•Condom negotiation-W
	•Activity: Unwritten rules about condoms-M
	•Safe sex negotiation and pleasure-M & W
	•Reducing risk-W
	•Making a commitment pledge-C
	•Homework (sex and relationship discussion)**
Workshop 2	•Gender roles-M
Module 1	•Activity: Act like a man/woman-M
	•Gender and HIV risk-W
	•Equality vs. equity-M
	•Activity: What is good sex?-M
	•Gender differences and sex-**3 slides
	•Unspoken rules and expectations-**2 slides
	•Unspoken expectations about sex**
	•What is sexy and safe?**
	•Activity: Pleasure brainstorm activity**
	•Staying a fit couple-C
	•Long-term partners at risk-W-2 slides
	•Speaker-listener technique (including DVD)-C-4 slides
	•Concerns about abuse-W-2 slides
	•Violence prevention-W
	•Rape and prevention with others-W-slides
Workshop 2	•Conflict-W-2 slides
Module 2	•Fair fighting-W-3 slides
	•Problem solving (including DVD)-C
	•Activity: Reducing your alcohol and other drug use as a couple**
	•Coping with stress-W
	•Neighborhood stressors**
	•Community strengths and resources**
	•Role models for children**
	•Concept of time in shebeens and risks**
	•Circle of safety**
	•Commitment pledge-C

Legend: ** =  New for this Couples Health CoOp; M =  Men as Partners; W =  WHC; C =  Project Connect.

## Discussion

This article describes a systematic process of adapting a couple-based HIV intervention for couples in South Africa. To the best of our knowledge, this is the first intervention to address the trifecta of substance use, gender-based violence, and sex risks for HIV within heterosexual couples in South Africa where the male partner uses alcohol.

The CHC intervention is novel in this context in that it not only addresses behavioral risks but also the relationship environment in which these risks occur, by equipping couples with communication and problem-solving skills to strengthen their relationships and family units. Furthermore, the intervention adaptation process described here provided the opportunity to integrate the world views of the couples and the expertise of local stakeholders to modify the core elements, the delivery style, and the structure of the intervention to enhance intervention fit to the cultural context.

The intervention elements were adapted and synthesized to respond to the HIV epidemic and context in South Africa and to ensure that the couples’ values and gender roles were integrated into the sessions and the delivery style. In the current research, the ADAPT framework was used to guide the adaptation process. Consequently, members of the community were consulted throughout all stages to elicit feedback on the various iterations of the intervention and to ensure that participants’ voices and ideas were salient in the actual intervention. In addition, a CCB assisted with the adaption process and in making linkages to referral services for program success. Previous adaptation studies of similar projects based on the original WHC also utilized such CCBs to help ensure the success of the adapted interventions [40].

Participants’ feedback from the pilot test as well as from the CCB on the feasibility and acceptability of this new intervention were constructive and positive. Their suggestions helped improve the CHC intervention and make it more acceptable for the targeted population. Previous literature has also indicated that community involvement during the development of an intervention helps create a supportive community context for the intervention when it is implemented [52].

The CCB also played an important role in other ways in the adapted intervention and its delivery. For example, CCB members helped to select and train community-peer leaders to develop and support positive role models for each target community, to conduct outreach to recruit potential participants for interventions, and to deliver the intervention within the community spaces.

During the adaptation, we ensured that we maintained all of the core elements of the WCWHC that were found to effective [53,54]. These provided the foundation for the new CHC intervention. However, we tailored the content of these elements to ensure that they met the needs of men who use alcohol and their female partners from poor communities in Cape Town.

In sum, the process of conducting an intervention adaptation and the use of contextually relevant interventions are important if it can begin to address the larger social determinants of HIV infection and transmission with male dominance and gender inequality and women’s lack of power in society.

## Conclusions

This article describes how an EBI to prevent HIV among vulnerable women was adapted to develop a multidimensional HIV prevention intervention for heterosexual couples in South Africa. The adaptation process highlighted the importance of working with couples to ensure that the intervention adequately addressed the local context and was culturally congruent with the values of the individuals in the target community. To achieve this congruence, we engaged with stakeholders, experts, and target participants in an iterative process. Although we started with evidence-based material and two programs that have been widely used in South Africa, the adapted intervention remains to be tested for efficacy to reduce alcohol and other drug use, sex risk behavior, and IPV among at-risk couples before recommendations can be made about its broader implementation. If shown to be efficacious, the CHC intervention may bring hope to communities in South Africa affected by IPV, substance use and sexual risk, all of which increase risk for HIV acquisition and transmission.

### Ethics statement

Ethics approval was granted by the Institutional Review Boards of RTI International and Stellenbosch University’s Faculty of Health Sciences. The trial (on which this study is based) is registered on ClinicalTrials.gov (Trial Registration Number: NCT01121692).

## Abbreviations

CCB: Community collaborative board
CDC: US centers for disease control and prevention
CHC: Couples’ health coop
EBI: Evidence-based intervention
IPV: Intimate partner violence
MAP: Men as partners
STI: Sexually transmitted infection
WCWHC: Western cape women’s health coop
WHC: Women’s health coop
